# Amelioration of diet-induced steatohepatitis in mice following combined therapy with ASO-Fsp27 and fenofibrate[S]

**DOI:** 10.1194/jlr.M077941

**Published:** 2017-09-05

**Authors:** Ananthi Rajamoorthi, Noemí Arias, Jeannine Basta, Richard G. Lee, Ángel Baldán

**Affiliations:** Edward A. Doisy Department of Biochemistry and Molecular Biology,* Saint Louis University, Saint Louis, MO; Department of Internal Medicine,† Saint Louis University, Saint Louis, MO; Center for Cardiovascular Research,** Saint Louis University, Saint Louis, MO; Liver Center,†† Saint Louis University, Saint Louis, MO; Cardiovascular Group,§ Antisense Drug Discovery, Ionis Pharmaceuticals, Carlsbad, CA

**Keywords:** steatosis, antisense therapy, fibrate, cell death-inducing DFFA-like effector C, peroxisome proliferator-activated receptor, nonalcoholic steatohepatitis, antisense oligonucleotide, fat-specific protein 27

## Abstract

Nonalcoholic fatty liver disease (NAFLD) is the leading cause of chronic liver disease. NAFLD progresses from benign steatosis to steatohepatitis (NASH) to cirrhosis and is linked to hepatocellular carcinoma. No targeted treatment is currently approved for NAFLD/NASH. We previously showed that fat-specific protein 27 (FSP27), a lipid droplet-associated protein that controls triglyceride turnover in the hepatocyte, is required for fasting- and diet-induced triglyceride accumulation in the liver. However, silencing *Fsp27* with antisense oligonucleotides (ASOs) did not improve hepatosteatosis in genetic nor nutritional mouse models of obesity. Herein, we tested the therapeutic potential of ASO-Fsp27 when used in combination with the PPARα agonist fenofibrate. C57BL/6 mice were fed a high-trans-fat, high-cholesterol, high-fructose diet for eight weeks to establish NASH, then kept on diet for six additional weeks while dosed with ASOs and fenofibrate, alone or in combination. Data show that ASO-Fsp27 and fenofibrate synergize to promote resistance to diet-induced obesity and hypertriglyceridemia and to reverse hepatic steatosis, inflammation, oxidative stress, and fibrosis. This multifactorial improvement of liver disease noted when combining both drugs suggests that a course of treatment that includes both reduced FSP27 activity and activation of PPARα could provide therapeutic benefit to patients with NAFLD/NASH.

Nonalcoholic fatty liver disease (NAFLD) is behind most cases of chronic liver disease in adults and children in Western societies (reviewed in Ref. 1, 2). NAFLD is a spectrum of liver disorders in the absence of significant alcohol intake that ranges from benign steatosis to steatohepatitis (NASH) to cirrhosis. NASH is characterized by hepatocyte ballooning and triglyceride (TAG) accumulation, inflammation, oxidative stress, and collagen deposition. Up to 25% of NASH patients develop hepatocellular carcinoma (1, 2). Liver failure from NAFLD and its complications are the third most common cause for liver transplantation and the twelfth leading cause of death in the United States. Together with dyslipidemia, central obesity, hypertension, and insulin resistance, NAFLD is a component of the metabolic syndrome, which confers increased risk for CVD (2). Several epidemiological studies suggest that up to 30% of Americans have some degree of NAFLD, but in obese, diabetic patients, these statistics climb to up to 80% (1, 2). Most projections suggest that the prevalence of NAFLD will continue to increase over the next few decades. Despite the growing threat of NAFLD, there remains a substantial lack of therapeutic tools to manage these patients.

Fat-specific protein 27 (FSP27), also known as CIDEC (cell death-inducing DFFA-like effector C) in humans, is a member of the CIDE family of lipid droplet-associated proteins that localizes to the surface of lipid droplet (LD) contact sites and promotes the formation of large, unilocular LDs in adipocytes by mediating the directional net transfer of lipids from small to large LDs. The three CIDE genes have distinct tissue distribution: CIDEA is abundant in brown adipose tissue, CIDEB in liver, and CIDEC in both white and brown adipose tissue (3, 4). Overexpression of *Fsp27* promotes the accumulation of larger LDs in several cell lines (5–8). Conversely, shRNA-mediated knockdown of *Fsp27* decreases LD size (9). Interestingly, *FSP27* is detected in fatty, but not in normal, livers (3, 10), and we showed that FSP27 promotes TAG accumulation in the hepatocyte under conditions of fasting and diet-induced hepatosteatosis and is under the transcriptional control of PPARα (10). Chow-fed *Fsp27^−^*^/−^ mice are lean, show small LDs in adipose tissue, and have lower plasma glucose and leptin, resulting in enhanced insulin sensitivity and resistance to diet-induced obesity (11, 12). Finally, a partially lipodystrophic patient was identified who carries a homozygous nonsense mutation in *FSP27* (13). The fact that hepatic *FSP27* is induced under pathologic conditions and correlates with lipid accumulation (3, 5, 10, 14, 15) suggests that FSP27 might be a therapeutic target for NAFLD/NASH patients. Consistent with that proposal, hepatocyte-specific *Fsp27^−^*^/−^ mice are protected from diet-induced hepatosteatosis (16), and a recent study showed that hepatic TAG contents were reduced in mice fed a high-fat diet (HFD) following a single infusion of an adenoviral-encoded shRNA against *Fsp27* (10, 16). Interestingly, this latter effect was enhanced by concomitant gavage with the synthetic PPARα agonist Wy14643 (10).

To test the therapeutic potential of FSP27-based therapies on the regression of preexistent NASH, herein we defined the consequences of long-term therapeutic silencing of *Fsp27* using generation 2.0 antisense oligonucleotides (ASOs), alone or in combination with the PPARα agonist fenofibrate, on the progression of diet-induced steatohepatitis. We hypothesized that PPARα-mediated increase in fatty acid oxidation is more efficient in promoting the hepatic clearance of lipids when used in combination with agents that prevent the storage of TAG in lipid droplets. C57BL/6 mice were dosed with ASOs, fenofibrate, or both after an 8-week feeding regimen with a high-trans-fat, high-cholesterol, high-fructose NASH diet, which was recently shown to induce hepatic steatosis, hepatocyte ballooning, inflammation, and fibrosis in mice (17–19), better modeling the histopathological features noted in the livers of NASH patients. Our data show that silencing *Fsp27* in the presence of fenofibrate results in the synergistic reduction of body weight, visceral adiposity, hepatic TAG accumulation, and liver inflammation, oxidative stress, and fibrosis, as well as changes in transcriptional programs regulating lipolysis, fatty acid utilization, and de novo lipogenesis in both liver and white adipose tissue (WAT). Importantly, we also report a major reduction in plasma VLDL-TAG upon silencing of *Fsp27*. Collectively, these data highlight the therapeutic potential of combination therapies that target both lipid storage and oxidation to manage NAFLD/NASH and reduce cardiovascular risk in patients.

## MATERIALS AND METHODS

### Chemicals

Chimeric 2′ methoxyethyl control (5′-CCTTCCCTGAAGGTTC­CTCC) and anti-Fsp27 (5′-CAGACTCTAATACCATTCAC) oligonucleotides were synthesized and purified, as has been described (20), suspended in saline, and stored at −20°C until used. A veterinarian at Saint Louis University filled a prescription for fenofibrate. Fenofibrate pills were pulverized, homogenized in saline, and used within 24 h.

### Mice

**Fig. 1.**
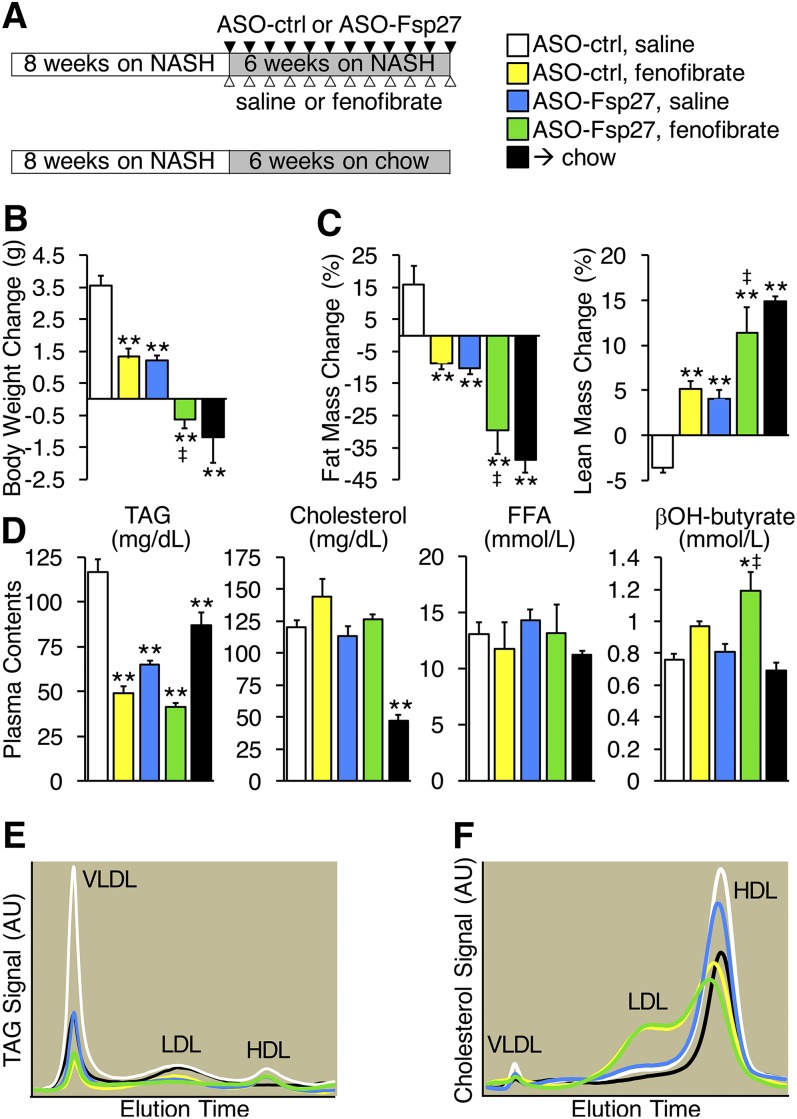
ASO-Fsp27 and fenofibrate synergistically reverse diet-induced obesity. A: Male C57BL/6 mice were fed NASH diet for 8 weeks and then kept on that diet or switched to chow for 6 additional weeks. Animals kept on the NASH diet were dosed with ASO-control (ctrl) or ASO-Fsp27 (IP), and saline or fenofibrate (oral gavage), as is described in the Materials and Methods section. B: Average body weight difference during the last 6 weeks. C: Percentage body composition for fat and lean mass, as measured by NMR. D: Plasma lipid and β-hydroxybutyrate contents at time of sacrifice. E: Plasma TAG lipoprotein profile from pooled samples, as was determined by FPLC. F: Plasma cholesterol lipoprotein profiles, as was measured by FPLC. Data are shown as means ± SEM (*n* = 7). *P ≤ 0.05, **P ≤ 0.01, compared with (ASO-ctrl, saline); ^‡^*P* ≤ 0.05, significant interaction between ASO-Fsp27 and fenofibrate treatments. Ctrl, control.

Animals were maintained in a 12-h/12-h light/dark cycle with ad libitum access to food and water. Six-week-old, male C57BL/6 mice (Jackson Laboratories, stock 000664) were fed NASH diet [Research Diets D09100301, containing 20% fat (65% trans-fat from Primex^®^ shortening; 20% saturated fat from lard), 1.8% cholesterol, and 20% fructose] for 8 weeks, then kept on NASH diet or crossed over to standard chow diet (PicoLab 5353) for 6 additional weeks. Where indicated, mice were dosed with 25 mg/kg ASO-ctrl or ASO-Fsp27 [Monday and Thursday, intraperitoneally (IP)], and 100 μl vehicle or 40 mg/kg fenofibrate (daily, oral gavage). ASO dose was chosen on the basis of our previous work (20). Fenofibrate dose is 0.7 times the maximum recommended human dose equivalent for mice, based on milligram per square meter of surface area and did not elicit liver carcinomas in long-term preclinical studies in rodents (US Food and Drug Administration [FDA] Pharmacology Review for fenofibrate, available at accessdata.fda.gov). For each experimental condition, *n* = 7. See **Fig. 1A** for details. Mice were sacrificed between 8:00 AM and 9:00 AM following an overnight fasting. Body composition was measured by NMR using an LF50 Analyzer (Bruker BioSpin, Billerica, MA). Animal studies were approved by the IACUC at Saint Louis University.

### Histology

Livers and epididymal WAT (eWAT) were fixed in 10% formalin, postfixed in 50% ethanol, and embedded in paraffin blocks. Sections (4 μm) were processed for hematoxylin and eosin, picrosirius (Polysciences; Warminister, PA), or F4/80 (Bio-Rad MCA497GA; 1:150 dilution; Raleigh, NC) staining. Under polarized light microscopy, type I and type II collagen fibers appear bright yellow/orange and green, respectively, in picrosirius-stained micrographs, and were quantified using ImageJ software, as has been described (21). F4/80-positive macrophages were stained as has been described (20). Adipocyte areas in hematoxylin/eosin-stained micrographs were calculated by using ImageJ software.

### Lipids

Tissue lipids were extracted into chloroform by a modified Folch method and resolubilized in water, as has been described (22). Specific lipid classes were quantified using enzymatic kits for triglycerides, total cholesterol, FFA, and phosphatidyl choline (Wako Chemicals, Richmond, VA). Results were normalized to total protein.

### Plasma analysis

Blood was collected from the inferior vena cava after an overnight fasting. Circulating lipids were quantified enzymatically in 5–20 μl of plasma with Wako kits. Ketone bodies were determined enzymatically in 10 μl of plasma with a kit from Cayman. Fast performance LC (FPLC) lipoprotein profiles from plasma samples were determined by a modified Column Lipoprotein Profile method. Briefly, plasma samples were pooled, diluted in saline (1:5 for cholesterol, 1:2 for triglycerides), and 40 μl injected into a Superose-6 column (GE Healthcare, Chicago, IL) by using elution buffer (saline, 2 mmol/l EDTA, 0.01% sodium azide, pH = 7.4) at 0.6 ml/min flow rate at 40°C. The eluate was mixed with cholesterol or TAG reagent (Pointe Scientific, Canton, MI) and incubated at 40°C in a 5-m KOT coiled reactor. The mixture entered a capillary spectrophotometer at 0.3 ml/min, and the signal was collected in real time using LC Solution software (Shimadzu, Kyoto, Japan).

### RNA

RNA was isolated from liver tissue with a Direct-zol RNA Miniprep kit (ZYMO Research, Irvine, CA) and from eWAT with RNeasy Lipid Tissue Mini Kit (Qiagen, Valencia, CA). The relative abundance of selected transcripts was determined by real-time quantitative PCR (qPCR), using PowerSybrGreen (Life Technologies, Carlsbad, CA) in a LightCycler LC480 instrument (Roche, Indianapolis, IN). Values were normalized to *36b4*, and relative expression was calculated with the ΔΔC_T_ method. Primer sets are available upon request.

### Protein

Liver proteins were extracted in 150 mmol/l NaCl, 1% NP-40, 0.1% SDS, 100 mmol/l Tris-HCl, pH 7.4, supplemented with protease inhibitors (Roche), and cleared by centrifugation at 4°C for 10 min at 10,000 *g*. Fifty micrograms of protein were resolved in either 4%–12% Bis-Tris or 3%–8% Tris-Acetate gels (Invitrogen), transferred to PVDF membranes, and probed with different primary and secondary antibodies in TBS-Tween20 containing 4% nonfat dry milk. Catalog numbers for each antibody and dilutions are provided in supplemental Table S1. Immune complexes were detected with SuperSignal West Pico chemiluminescent substrate (Pierce) and normalized to vinculin. Protein carbonylation was assessed in 20 μg of liver extract using the OxyBlot Protein Oxidation Detection kit (Millipore), following the manufacturer’s instructions.

### Statistics

Data are shown as mean ± SEM. Differences were analyzed by two-way ANOVA followed by posthoc Bonferroni’s test. A *P* value of ≤ 0.05 was considered statistically significant.

## RESULTS

### ASO-Fsp27 and fenofibrate synergize to reverse diet-induced obesity

To determine the effects of a combined therapy (Fsp27 silencing and PPARα activation) on diet-induced steatohepatitis, we fed C57BL/6 mice a high-trans-fat, high-cholesterol, high-fructose diet that has been shown to result in severe hepatosteatosis and mild hepatic inflammation and fibrosis (18, 19, 21, 23). After 8 weeks on the NASH diet, mice were randomized into four groups and kept on the same diet for 6 additional weeks while dosed with ASO-ctrl or ASO-Fsp27 and saline or fenofibrate (Fig. 1A). An additional age-matched group was fed the NASH diet for 8 weeks, then switched to chow (Fig. 1A). Data in Fig. 1B and supplemental Fig. S1A show that control mice on the NASH diet gained weight during the last 6 weeks of the experiment, as opposed to those switched to chow. Treatment with fenofibrate or ASO-Fsp27 alone resulted in significantly less weight gain, in comparison with NASH-fed control mice. Combining both treatments, however, resulted in weight loss similar to that in mice switched to chow. Consistent with these data, analysis of body composition by NMR revealed a significant decrease in fat mass upon treatment with either fenofibrate or ASO-Fsp27, in comparison with control NASH-fed mice, but combined treatment led to a synergistic reduction in fat mass, again similar to that in mice switched to chow diet (Fig. 1C). As was expected, lean mass contents reverse-mirrored the data on fat contents (Fig. 1C). Importantly, these changes in body weight and body composition in NASH-fed mice occurred despite the fact that caloric intake was not significantly different among groups (supplemental Fig. S1B).

As was expected, fenofibrate robustly reduced plasma TAG (Fig. 1D and supplemental Fig. S1C). Silencing *Fsp27* also decreased circulating TAG, similar to fenofibrate, but no synergistic effect was noted with the combined therapy (Fig. 1D and supplemental Fig. S1C). In contrast, neither fenofibrate nor ASO-Fsp27 changed total cholesterol nor FFA in plasma (Fig. 1D). Notably, β-hydroxy-butyrate was elevated in plasma samples of mice dosed with both ASO-Fsp27 and fenofibrate (Fig. 1D), suggesting accelerated hepatic fatty acid oxidation. Plasma lipids were also analyzed by FPLC to determine lipoprotein profiles. The data confirmed the changes in VLDL-TAG (Fig. 1E) and revealed a large change in the HDL/LDL distribution of cholesterol in response to fenofibrate (Fig. 1F).

### Reduced visceral adiposity in NASH-fed mice treated with ASO-Fsp27 or fenofibrate

Consistent with the NMR data above, supplemental Fig. S2A, B shows a robust reduction in eWAT in NASH-fed mice dosed with either fenofibrate or ASO-Fsp27 and a trend for further reduction in mice receiving the double treatment. These data are consistent with our previous report of a robust decrease in visceral fat pads after sustained silencing of *Fsp27* in HFD-fed mice (20). Lipid analysis of eWAT samples from NASH-fed mice revealed that TAG contents mirrored the changes in tissue mass, whereas cholesterol levels were highly variable within each experimental group, and phosphatidylcholine contents remained unchanged (supplemental Fig. S2C). Histologically, white adipocytes were larger in NASH-fed mice than were those in mice switched to chow, and reduced average cell size was markedly noticeable in samples from mice dosed with both ASO-Fsp27 and fenofibrate (supplemental Fig. S2D).

A selected panel of lipid-related transcripts was analyzed in the same eWAT samples (supplemental Fig. S3). Data show that ASO-Fsp27 was effective in reducing *Fsp27* expression (80%–95%). The expression of canonical PPARα targets (*Fsp27*, *Cpt1a*, *Mcad*, *Acox*), however, remained largely unaltered by the fenofibrate treatment alone. This apparent lack of effect of fenofibrate could be the consequence of the relatively low levels of PPARα in the adipocyte; alternatively, perhaps diet-derived PPARα ligands already provide maximal induction of these transcripts in adipose tissue. In contrast, ASO-Fsp27, or a combination of ASO-Fsp27 and fenofibrate, resulted in the decrease of multiple lipid droplet-associated (*Cidea*, *Plin1*) and lipogenic (*Srebp1c*, *Fasn*, *Scd1*) transcripts. The large macroscopic reduction in eWAT in mice dosed with ASO-Fsp27, fenofibrate, or both are likely explained by the previously reported reduction in lipid storage, synthesis, or accelerated mobilization of FFA, or all of these, from the lipid droplet upon loss of adipocyte FSP27 activity (24) or following PPARα activation (25).

### Combined therapy with ASO-Fsp27 and fenofibrate ameliorates diet-induced steatohepatitis

**Fig. 2.**
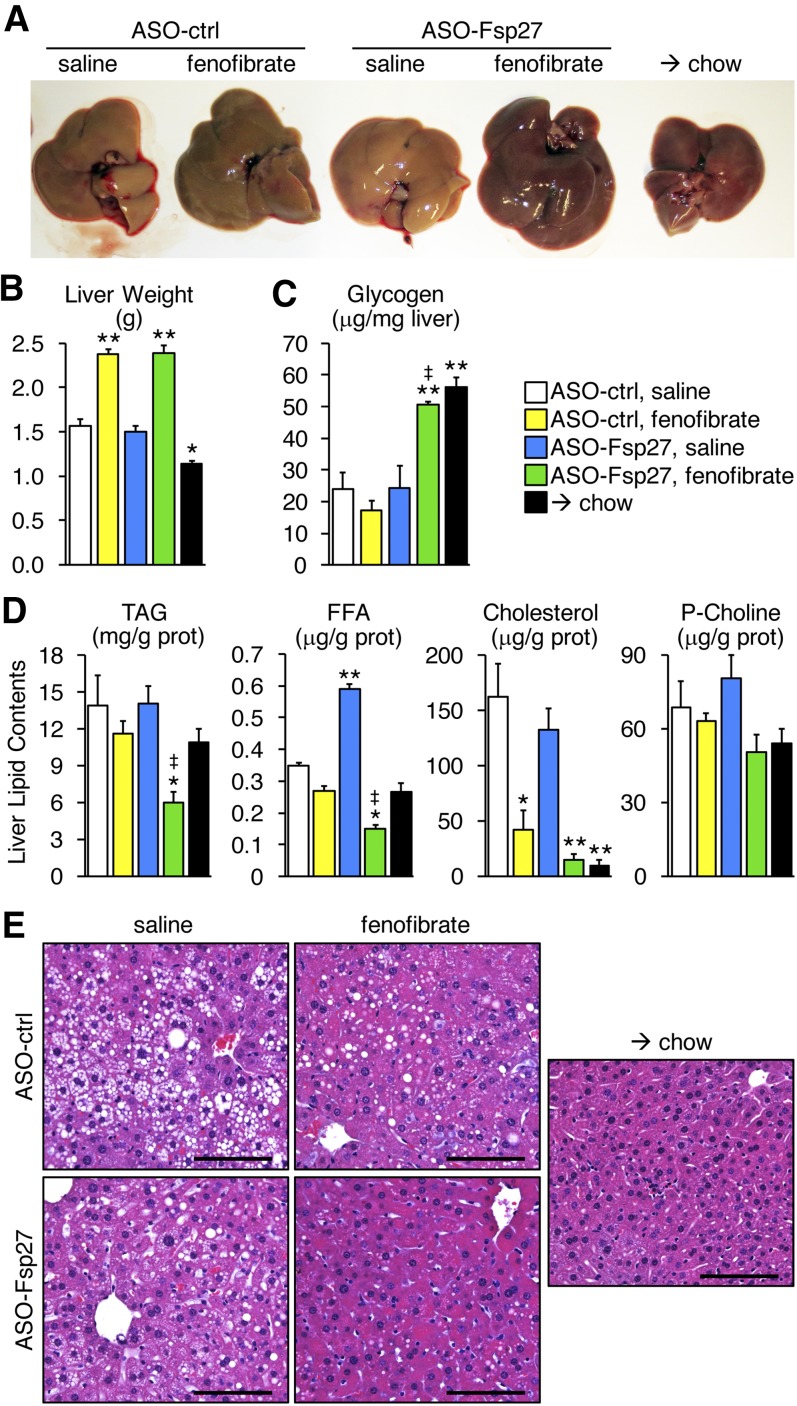
ASO-Fsp27 and fenofibrate synergistically reverse diet-induced hepatosteatosis. A: Representative macroscopic appearance of the livers in each experimental group. B: Liver weights. C: Hepatic glycogen contents. D: Hepatic lipid contents. E: Representative hematoxylin and eosin staining of paraffin embedded sections. Scale bars represent 100 μm. Data are shown as means ± SEM (*n* = 7). *P ≤ 0.05, **P ≤ 0.01, compared with (ASO-ctrl, saline); ^‡^*P* ≤ 0.05, significant interaction between ASO-Fsp27 and fenofibrate treatments. Prot, protein.

As was expected in mice (26), fenofibrate treatment resulted in hepatomegaly (**Fig. 2A, B**). The livers of mice that received the combined therapy were consistently darker than were those in the other experimental groups (Fig. 2A), suggesting that the lipid contents might be reduced. Data in Fig. 2C show that hepatic glycogen contents in mice dosed with ASO-Fsp27 and fenofibrate were elevated, in comparison with the rest of NASH-fed animals, and similar to those from mice switched to chow. Enzymatic assays in lipid extracts confirmed the robust decrease in hepatic TAG contents in mice treated with both ASO-Fsp27 and fenofibrate (Fig. 2D). Neither treatment alone reduced steatosis (Fig. 2D). Unexpectedly, the switch from NASH diet to chow resulted only in relatively modest changes in liver weight and steatosis (Fig. 2A, B). The amounts of FFA were increased in livers from mice treated with ASO-Fsp27 (Fig. 2D), which is consistent with previous reports of defective expression of hepatic PPARα fatty acid oxidation targets upon sustained loss of FSP27 (20, 27). In contrast, concomitant ASO-Fsp27 and fenofibrate sharply reduced liver FFA contents (Fig. 2D). The amounts of phosphatidyl choline remained unchanged among the experimental groups (Fig. 2D). Reduced amounts of hepatic cholesterol were noted in mice treated with fenofibrate alone but not with ASO-Fsp27 alone; there was a trend for further decrease in mice receiving the combined therapy, but it did not reach statistical significance (Fig. 2D). The differences in TAG contents were paralleled by profound tissue architectural changes (Fig. 2E and supplemental Fig. S4). In control NASH-fed mice, extensive areas of macrovesicular steatosis and hepatocyte ballooning were noted. These were partially, but not completely, reduced in the livers of mice receiving either of the individual treatments, but foamy hepatocytes were noticeable. Foamy cells, but not areas of macrovesicular steatosis, were noted in mice switched to chow. In contrast, livers from mice receiving both treatments looked largely normal, and few foamy hepatocytes were present.

**Fig. 3.**
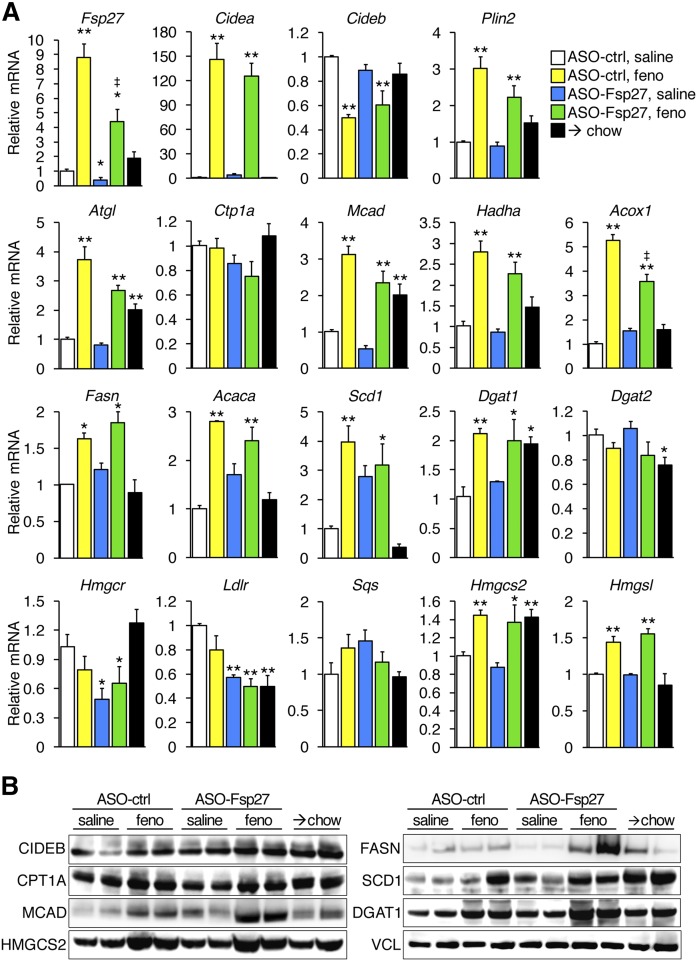
Hepatic relative expression of selected lipid-related genes. A: RNA contents were analyzed by qPCR. B: Immunoblots in the same livers. Data show the efficiency of *Fsp27* silencing, and the induction of bona fide PPARα targets by fenofibrate. Transcript data are shown as mean ± SEM (*n* = 5). *P ≤ 0.05, **P ≤ 0.01, compared with (ASO-ctrl, saline); ^‡^*P* ≤ 0.05, significant integration between ASO-Fsp27 and fenofibrate treatments. Feno, fenofibrate.

Data in **Fig. 3** show the relative expression of selected lipid-related transcripts and proteins in the liver. As we reported previously (10), fenofibrate induces the expression of hepatic *Fsp27*. The ASO treatment significantly reduced the levels of *Fsp27* in saline-treated mice and partially blunted its fenofibrate-stimulated induction. Treatment with fenofibrate alone simultaneously resulted in the induction of transcripts involved in lipid mobilization and oxidation (*Atgl*, *Acox*, *Mcad*, *Hadha*), lipogenesis *(Fasn*, *Acaca*, *Scd1*), and storage (*Cidea*, *Plin2*), consistent with previous reports (28). In contrast, ASO-Fsp27 treatment alone did not significantly change the expression of lipid droplet-associated, lipolytic, or oxidative mRNAs but resulted in the significant reduction in *Hmgcr* and *Ldlr* transcripts. In general, protein data largely paralleled the changes in mRNA and show that classic PPARα targets are indeed induced in the livers of fenofibrate-treated mice. The only discrepancies noted between mRNA and protein data were the apparent stabilization of SCD1 protein in mice switched to chow and the flat levels of CIDEB protein, both likely due to posttranslational regulation. Despite the robust consequences of combined therapy on hepatic steatosis described above, we did not identify obvious synergistic effects on gene expression.

**Fig. 4.**
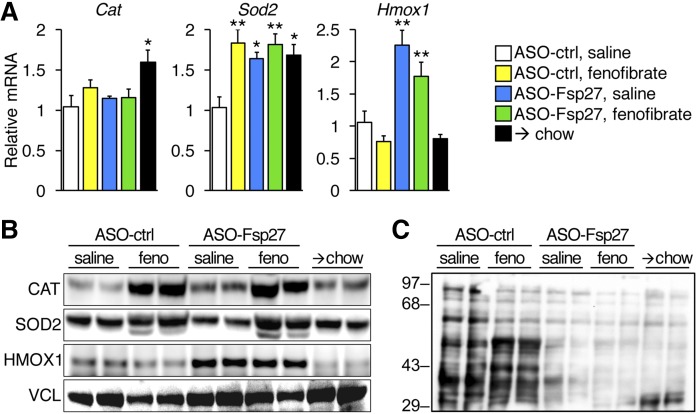
Treatment with ASO-Fsp27 reduces hepatic oxidative stress. A: Relative expression of catalase, superoxide dismutase-2, and heme oxygenase-1, as measured by qPCR. B: Immunoblots for the same proteins using two individual livers per group. C: Protein carbonylation in liver homogenates from the same mice. Transcript data are shown as means ± SEM (*n* = 5). *P ≤ 0.05, **P ≤ 0.01, compared with (ASO-ctrl, saline).

**Fig. 5.**
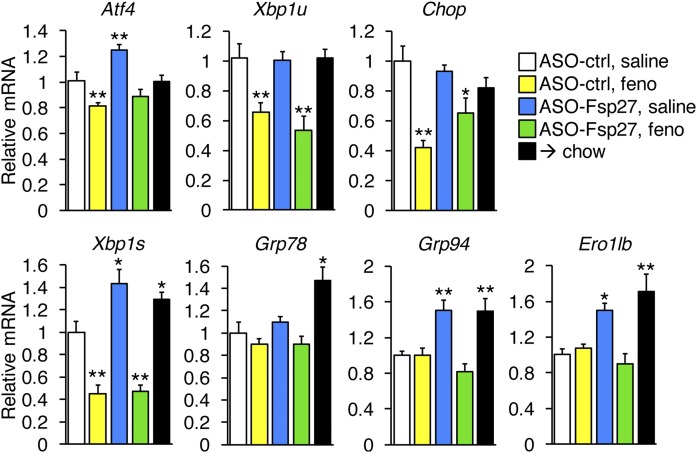
Fenofibrate and ASO-Fsp27 promote distinct changes in ER stress pathways. Disruption of ER homeostasis can trigger the activation of three ER-associated proteins: PERK, IRE1a, and ATF6. The associated signaling cascade events ultimately promote the transcription of specific genes. Data show hepatic relative expression of selected ER stress-induced transcripts, as measured by qPCR. Data are shown as means ± SEM (*n* = 5). *P ≤ 0.05, **P ≤ 0.01, compared with (ASO-ctrl, saline).

Lipid catabolism is intimately associated with the production of toxic reactive oxygen species (ROS) and endoplasmic reticulum (ER) stress, which in turn correlate with the severity of steatohepatitis in patients and animal models. As indirect evidence of ROS abundance, we measured both the expression of antioxidant genes (**Fig. 4A, B**) and protein carbonylation (Fig. 4C). Data show that mRNA and protein levels of these genes were not always linked. Thus, fenofibrate stimulated the accumulation of catalase protein (but not mRNA) and superoxide dismutase-2 (mRNA and protein), whereas ASO-Fsp27 treatment increased *Sod-2* mRNA (but not protein) and significantly increased heme oxygenase-1 (mRNA and protein). In any case, a broad reduction in ROS was noted in the livers of mice dosed with ASO-Fsp27, alone or in combination with fenofibrate, as measured by protein carbonylation, which was similar to that in mice switched to chow (Fig. 4C). Interestingly, the effects of fenofibrate on protein carbonylation varied: higher (lower) molecular bands were in general weaker (stronger) than were those in NASH control livers (Fig. 4C). Next, we monitored hepatic ER stress by measuring the expression of canonical transcripts that are induced by each of the arms of the unfolded protein response (**Fig. 5**). Data show that fenofibrate significantly reduced PERK-dependent signaling, as was evidenced by the decrease in the ATF4 transcriptional targets *Chop* and unspliced *Xbp1*. In contrast, ASO-Fsp27 induced the expression of downstream effectors of IRE1α, such as spliced *Xbp1*, and XBP1s targets *Grp94* and *Ero1lb*. Livers from mice dosed with the combined therapy showed reduced PERK signaling but no changes in IRE1α targets. Unexpectedly, switching the mice to chow also resulted in changes in ER stress-related transcripts that were similar to those in ASO-Fsp27-treated animals. Taken together, these data suggest that ASO-Fsp27 treatment reduces oxidative stress and activates ER pathways that mimic the switch to chow, whereas fenofibrate has a limited impact on oxidative stress and reduces PERK signaling.

**Fig. 6.**
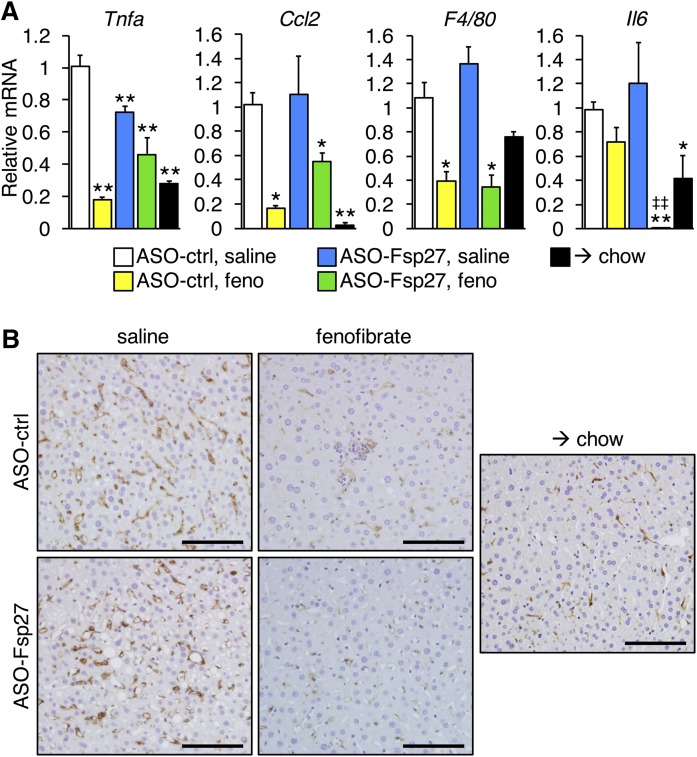
Fenofibrate and ASO-Fsp27 reduce hepatic inflammation. A: Relative expression of selected proinflammatory transcripts. B: Representative micrographs of F4/80 immunohistochemical staining of paraffin-embedded liver sections. Dark brown precipitate indicates F4/80-positive macrophages. Scale bars represent 100 μm. RNA data are shown as means ± SEM (*n* = 5). *P ≤ 0.05, **P ≤ 0.01, compared with (ASO-ctrl, saline); ^‡‡^*P* ≤ 0.01, significant interaction between ASO-Fsp27 and fenofibrate treatments.

**Fig. 7.**
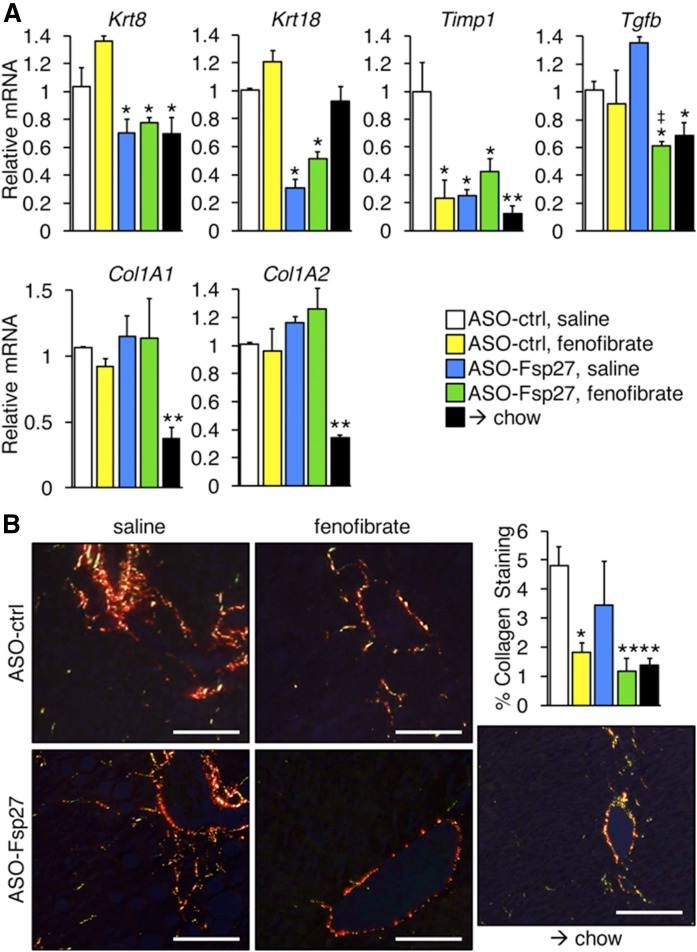
Fenofibrate and ASO-Fsp27 reduce hepatic fibrosis. A: Relative expression of selected fibrotic transcripts. B: Representative polarized light micrographs of paraffin-embedded sections stained with Picrosirius red. Yellow-to-orange signal reveals the presence of zone 3 perisinusoidal collagen fibers, especially in the ASO-ctrl group. Scale bars represent 100 μm. Percentage collagen area was estimated from 10 random fields per experimental group. Data are shown as means ± SEM (*n* = 5). *P ≤ 0.05, **P ≤ 0.01, compared with (ASO-ctrl, saline); ^‡^*P* ≤ 0.05, significant interaction between ASO-Fsp27 and fenofibrate treatments.

Finally, we also examined the relative expression of selected transcripts involved in hepatic inflammation and fibrosis (**Figs. 6**, ** 7**). Data show that fenofibrate was the main driver promoting the decrease of several proinflammatory markers (*Tnfα*, *Ccl2*, *F4/80*); however, ASO-Fsp27 alone also reduced *Tnfα*, and combined therapy resulted in a synergistic decrease in interleukin-6 (Il-6) (Fig. 6A). The anti-inflammatory effects of fenofibrate were also noticeable by immunohistochemical analysis of macrophage infiltration in liver sections (Fig. 6B). Long-term exposure to fibrates is hepatocarcinogenic in rodents but not in humans (26). At the relatively low dose we used, we found no signs of tumors, as assessed in multiple tissue section micrographs and by the undetectable levels of the typical markers of hepatocellular carcinoma *Afp* and *H19* (data not shown). The expression of several markers of fibrosis that include keratin and collagen transcripts was reduced following the switch to chow diet (Fig. 7A). Additionally, increased collagenolytic activity in the animals switched to chow was inferred from the sharp decreased expression of *Timp1* (Fig. 7A). The effects of the treatments, alone or in combination, on the abundance of these transcripts varied, though. Thus, ASO-Fsp27, but not fenofibrate, promoted a decrease in keratins 8 and 18; combined treatment led to decreased expression of *Tgfβ*; and other transcripts were unaffected (Fig. 7A). Finally, we examined the amount of fibrosis in liver sections by staining with Picrosirius red (Fig. 7B). Data show that the effects of ASO-Fsp27 on collagen deposition were largely variable among mice, and either fenofibrate or combination therapy reduced collagen contents to a similar extent as did diet crossover.

Collectively, data in Figs. 2–7 suggest that activation of PPARα and sustained silencing of *Fsp27* in mice fed a NASH diet result in improved liver macroscopic and histological appearance, as well as in reduced hepatic TAG contents, oxidative stress, inflammation, and fibrosis.

## DISCUSSION

**Fig. 8.**
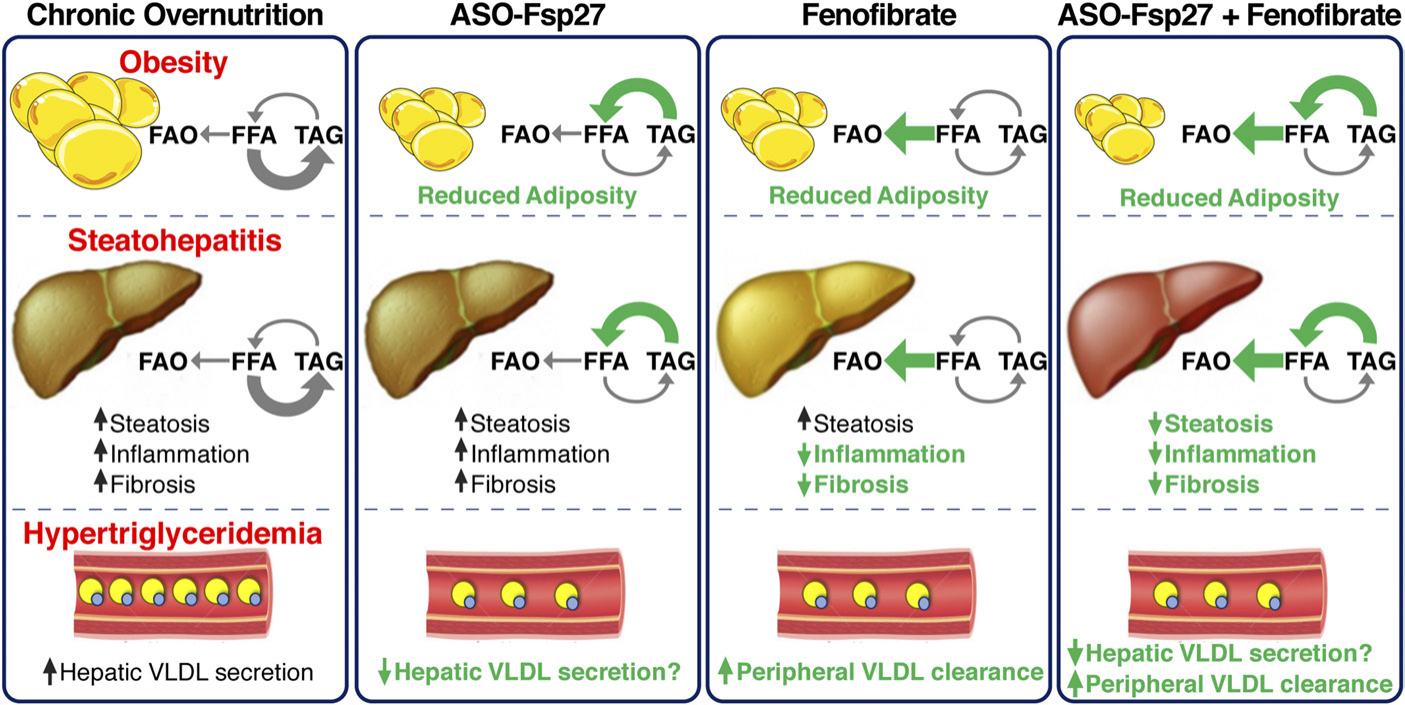
ASO-Fsp27 and fenofibrate combined therapy. Chronic overnutrition results in obesity, fatty liver disease, and dyslipidemia. Under those conditions, therapeutic reduction of FSP27 (e.g., by silencing with ASOs) promotes enhanced lipolytic activity at the surface of the LD. In the hepatocyte, for unknown reasons the resulting increase in FFA is not efficiently oxidized in the mitochondria, leading to no substantial changes in hepatic steatosis. In contrast, in the adipose tissue of the same animals, FFA are likely secreted into circulation, leading to significantly reduced adiposity. Silencing FSP27 also reduces circulating VLDL-TAG, probably by decreasing the pool of TAG available for secretion. Activation of PPARα (e.g., by fenofibrate) has well-recognized anti-inflammatory, antifibrotic, and hypotriglyceridemic effects but cannot efficiently promote increased lipid oxidation in the hepatocyte. Presumably under those conditions, LD-associated, PPARα targets (FSP27, PLIN2, PLIN5) limit the availability of substrates toward the mitochondria. Combination therapy including ASO-Fsp27 and fenofibrate allows the mobilization of LD-stored lipids and enhances their oxidation. In the liver, these synergistic effects normalize TAG contents and further reduce markers of inflammation and fibrosis. FAO, fatty acid oxidation.

Studies in mice (10, 15, 29) and humans (30, 31) showed that under pathological conditions, the expression of hepatic *CIDEC/Fsp27* is highly increased and correlates with the degree of hepatosteatosis. On the basis of those data, it was hypothesized that *CIDEC/Fsp27* might be exploited therapeutically to alleviate diet-induced NAFLD/NASH. Consistent with that proposal, an adenoviral-mediated shRNA against *Fsp27* acutely reversed hepatic steatosis induced by fasting (10) or HFD (10, 16). A 5-week treatment with ASO-Fsp27 reduced visceral adiposity and restored insulin sensitivity in adipose tissue, liver, and muscle in both HFD-fed C57BL/6 mice and chow-fed *ob/ob* mice (20). In these latter long-term studies, however, ASO-Fsp27 treatment did not reduce liver steatosis (20), despite the sharp decrease noted in previous short-term studies (10). These differences might be related to the global silencing effects of ASOs (adipose tissues and liver), compared with the liver-restricted silencing of adenoviral-encoded shRNAs. Thus, the partial loss of *Fsp27* expression in both WAT and liver in ASO studies limits the interpretation of the liver phenotype. Interestingly, the expression of PPARα targets was found to be significantly reduced in the livers of the ASO-Fsp27-treated mice (10) and in long-term HFD-fed *Fsp27*^−/−^ mice (27), suggesting that efficient β-oxidation capacity was reduced in the hepatocyte after sustained loss of FSP27 activity. The reasons behind the decrease in PPARα oxidative targets in those livers are obscure, but the increased amounts of FFA in the livers of mice treated with ASO-Fsp27 we report herein are consistent with that proposal. Importantly, studies reported both epigenetic (32) and posttranscriptional (33, 34) silencing events that reduce the expression of hepatic PPARα in mice and humans with NASH. Nonetheless, here we tested the hypothesis that exogenous provision of a PPARα agonist (e.g., fenofibrate) would rescue the activity of oxidative genes and promote the accelerated mobilization of lipids in the liver. Previous mechanistic studies in primary hepatocytes in our laboratory showed that concurrent loss of FSP27 and provision of an exogenous PPARα agonist maximize lipid turnover and mobilization toward mitochondrial oxidation (10). The in vivo data herein expand those initial results and further demonstrate that silencing of *Fsp27* and fenofibrate synergistically reduce body weight gain, visceral adiposity, hypertriglyceridemia, liver oxidative stress, and steatohepatitis in a diet-induced mouse model of NASH (**Fig. 8**). The observation that mice receiving the combined therapy lost weight despite having similar caloric intake than did the other groups on a NASH diet strongly supports a model of increased energy expenditure, likely through accelerated fatty acid oxidation. Elevated ketone bodies in plasma of these same mice provides further evidence for this model. Importantly, we also report a major reduction in plasma VLDL-TAG following *Fsp27* silencing. We speculate that loss of FSP27 activity reduces the pool of TAG available for VLDL maturation and secretion. Future studies will test this proposal.

There are currently no FDA-approved drugs for targeted treatment of NASH. However, weight loss (usually accomplished by caloric restriction and exercise, or following bariatric surgery) significantly improves hepatic steatosis, inflammation, and fibrosis in obese patients (2). It is possible that the significant reduction in overall adiposity noted in our mice contributed to the amelioration of steatohepatitis in the ASO-Fsp27 plus fenofibrate group, independently of direct effects of silencing *Fsp27* and inducing PPARα activation in the liver. However, weight loss is unlikely to be the sole mechanism behind liver improvement, given that ASO-Fsp27 or fenofibrate alone also decreased adiposity but did not ameliorate hepatosteatosis. The metabolic cross-talk between adipose tissues and liver is a limitation of our ASO approach, though, and separating the contributions of FSP27 in each tissue to the whole-body phenotype, with or without fenofibrate, will require the generation of tissue-specific *Fsp27*^−/−^ mice. Mechanistically, the reduction in liver TAG following combined treatment is consistent with our previous data in primary hepatocytes, in which concomitant shRNA-mediated knock-down of *Fsp27* and PPARα activation with GW7647 resulted in enhanced TAG turnover and mitochondrial β-oxidation (10). Likely the accelerated lipolysis of TAG at the surface of the lipid droplet following loss of FSP27 activity facilitates the release and delivery of substrates to the mitochondria. Similar to our studies in isolated hepatocytes (10), the decrease in hepatic TAG contents in our mice occurred in the absence of apparent changes in the expression of oxidative genes, compared with livers from mice dosed with fenofibrate only. We hypothesize that FSP27 acts as a brake for PPARα-stimulated fatty acid oxidation by limiting the release of substrates from the lipid droplet toward β-oxidation, without changing the amounts or activities of fatty acid oxidative enzymes.

Potential unwanted consequences of accelerated fatty acid utilization are both an increase in cytotoxic ROS and the induction of ER stress. Oxidative stress rises during the progression of liver disease and correlates with the severity of NASH (35, 36). PPARα has been shown to regulate redox signaling in oxidative tissues by promoting the expression of mitochondrial and peroxisomal oxidative and cytoprotective genes (37). Our data show that fenofibrate increased both mRNA and protein expression of the mitochondrial enzyme superoxide dismutase-2, which was previously reported as a direct PPARα target. The amounts of the peroxisomal enzyme catalase were also raised by fenofibrate, presumably due to the peroxisomal proliferation that PPARα agonists elicit in the murine liver, given that mRNA levels remained unaltered. On the other hand, treatment with ASO-Fsp27 raised both mRNA and protein levels of the microsomal enzyme Heme oxygenase-1 (HMOX-1). Accumulating evidence suggests that HMOX-1 is induced by a variety of nonheme products and signaling pathways and plays a key role in mediating protection against oxidative tissue injury. Importantly, experiments using chemical inhibitors and adenoviral overexpression suggest that induction of HMOX-1 is critical for protection against diet- (38–40) or ethanol- (41–43) induced steatohepatitis in mice. Our data also show that protein carbonylation, the most prominent type of protein modification due to oxidative stress (44), was markedly reduced in the livers of mice dosed with ASO-Fsp27, with or without fenofibrate, and similar to that in animals switched to chow. These oxyblot and gene expression results are consistent with those in a report showing reduced ethanol-induced ROS in liver-specific *Fsp27*^−/−^ mice (45). Together, our results suggest that silencing FSP27 promotes a strong protective response against hepatic oxidative stress, although the molecular mechanisms that link changes in FSP27 to ROS inactivation are not immediately evident.

A well-described consequence of increased levels of reactive oxygen species is endoplasmic reticulum stress and the activation of the unfolded protein response (46). We monitored the activation of hepatic PERK-eIF2α-ATF4, IRE1α-XBP1, and ATF6 pathways by measuring the expression of key effector mRNAs in each arm of the unfolded protein response. Our data show that fenofibrate reduced PERK signaling without affecting IRE1α and ATF6 pathways. The protective effects of PPARα activation on PERK-dependent ER stress in the hepatocyte had been previously described (47). In contrast, ASO-Fsp27 induced the expression of downstream effectors of IRE1α and ATF6. Livers from mice dosed with the combined therapy showed reduced PERK signaling but no changes in IRE1α/ATF6 targets. Just as with ROS metabolism, the molecular mechanisms by which lipid turnover in the LD affect ER stress signaling remain to be established. The unexpected observation that mice fed chow displayed changes in ER stress-related transcripts similar to those in ASO-Fsp27-treated animals strongly suggests that the induction of those pathways are likely adaptive and hepatoprotective. We speculate that the combined therapy allows the concomitant induction of these beneficial pathways (by ASO-Fsp27) and the reduction in PERK signaling (by fenofibrate).

Recent studies suggest that the anti-inflammatory and antifibrotic effects of fibrates in the mouse liver are due to the direct transcriptional repression of inflammatory and fibrotic genes and are not dependent on transactivation of lipid-related genes (48, 49). Data presented herein are in part consistent with that model: the synergistic effects of ASO-Fsp27 and fenofibrate were mostly noted in terms of decreasing hepatic steatosis, and the changes in inflammation and fibrosis were largely driven by the fibrate. However, ASO-Fsp27 alone or in combination with fenofibrate also modestly affected the expression of selected inflammatory and fibrotic transcripts and substantially reduced hepatic oxidative stress.

PPARα agonists such as fenofibrate have been used clinically for several decades and provide a well-established cardiovascular benefit to patients with hypertriglyceridemia (50, 51). The hypolipidemic effects of fibrates have been mostly attributed to increased peripheral LPL-mediated clearance of triglyceride-rich lipoproteins (52, 53). Reports from *Pparα*^−/−^ mice and studies with synthetic agonists in a variety of experimental murine models revealed that PPARα orchestrates fatty acid synthesis, storage, and oxidation in the hepatocyte (28). Interestingly, we observed a significant reduction in hepatic cholesterol contents in mice dosed with fenofibrate, which is consistent with a previous report of PPARα-dependent changes in SREBP-2 signaling (54). The effects of fibrates on body weight and energy expenditure are well documented, with studies showing resistance to diet-induced weight gain and induction of thermogenesis and “browning” in subcutaneous white adipose tissue in rodents (55). However, the effects of fibrates on body weight have not been well characterized in human clinical studies. Our data suggest that the combination of *Fsp27* silencing and PPARα activation may promote weight loss in overweight/obese populations. Because several canonical PPARα targets promote increased fatty acid oxidation, it was speculated that patients with a fatty liver would benefit from a treatment with fibrates. However, the functional role of PPARα in NAFLD/NASH remains controversial. Whole-body and hepatocyte-specific Pparα^−/−^ mice develop hepatosteatosis, inflammation, and fibrosis over the course of 1 year on a standard diet, as well as on short exposure to HFD (56, 57). Additionally, fibrates have been shown to reduce or prevent liver disease in diet-induced mouse models of steatohepatitis (58, 59). These data indicate a strong therapeutic potential for targeting hepatic PPARα in NAFLD/NASH. However, other studies reported an increase in liver TAG contents following PPARα activation (60, 61). Finally, most, but not all, studies on fibrates and human NASH failed to show improved liver histology or decreased liver TAG contents (reviewed in Ref. 62).

The lack of a strong therapeutic benefit of fibrates in NASH clinical studies is perplexing. We hypothesize that lipid droplet-associated proteins such as FSP27 pose a lipolytic barrier that prevents efficient lipid mobilization in the hepatocyte, even under conditions in which fatty acid oxidative genes are induced by PPARα agonists. Interestingly, *Fsp27* (10), *Plin2* (63), and *Plin5* (64) are all bona fide direct PPARα targets. Additionally, fibrates also promote hepatic lipid synthesis in wild-type, but not in Pparα^−/−^, mice (28, 65). The promotion of seemingly antagonistic transcriptional programs (oxidation, storage, synthesis) by PPARα likely reflects the need to prevent lipotoxic events in the hepatocyte. Our data suggest that specific PPARα modulators (SPPARMs) that preferentially induce the expression of oxidative targets, rather than lipogenic or LD-associated targets, might be used therapeutically to reduce TAG deposition in the livers of NAFLD/NASH patients. Indeed, several SPPARMs are currently at different stages of development for the treatment of dyslipidemias and NAFLD/NASH (66, 67).

Obesity, hypertriglyceridemia, and fatty liver diseases are intimately connected and are independent risk factors for CVD. The data in this study suggest that the synergistic effects of *Fsp27* silencing and PPARα activation could be exploited therapeutically to reduce diet-induced body weight gain, visceral obesity, hypertriglyceridemia, and steatohepatitis.

## Supplementary Material

Supplemental Data
