# Supplementary material for: Amelioration of diet-induced steatohepatitis in mice following combined therapy with ASO-Fsp27 and fenofibrate
Source: J Lipid Res. 2017 Sep 5;58(11):2127–38. doi: 10.1194/jlr.M077941 (PMC5665668; doi:10.1194/jlr.M077941)
Supplement: Supplemental Data [file supp_58_11_2127__index.html]

Amelioration of diet-induced steatohepatitis in mice following combined therapy with ASO-Fsp27 and fenofibrate — Amelioration of diet-induced steatohepatitis in mice following combined therapy with ASO-Fsp27 and fenofibrate — Supplemental Data 

# Amelioration of diet-induced steatohepatitis in mice following combined therapy with ASO-Fsp27 and fenofibrate

## Supplemental Data

- Supporting Information (.pdf, 1.7 MB) - Supplemental Information
