# Supplementary material for: Amelioration of diet-induced steatohepatitis in mice following combined therapy with ASO-Fsp27 and fenofibrate
Source: J Lipid Res. 2017 Sep 5;58(11):2127–38. doi: 10.1194/jlr.M077941 (PMC5665668; doi:10.1194/jlr.M077941)
Supplement: Supplemental Data [file 10.1194_M077941_jlr.M077941-1.pdf]

SUPPORTING INFORMATION

Amelioration of diet-induced steatohepatitis in mice following combined therapy with ASO-Fsp27 and fenofibrate

Ananthi Rajamoorthi[1], Noemí Arias[1], Jeannine Basta[2], Richard G. Lee[3], Ángel Baldán[1,4,5]

[1]Edward A. Doisy Department of Biochemistry & Molecular Biology, [2]Department of Internal Medicine, Saint Louis University, Saint Louis, MO 63104; [3]Cardiovascular Group, Antisense Drug Discovery, Ionis Pharmaceuticals, Carlsbad, CA 92010; [4]Center for Cardiovascular Research, and [5]Liver Center, Saint Louis University, Saint Louis, MO 63104

Running Title: ASO-FSP27 and fibrate therapy in fatty liver disease

Supplemental Figures S1–4

Supplemental Table S1

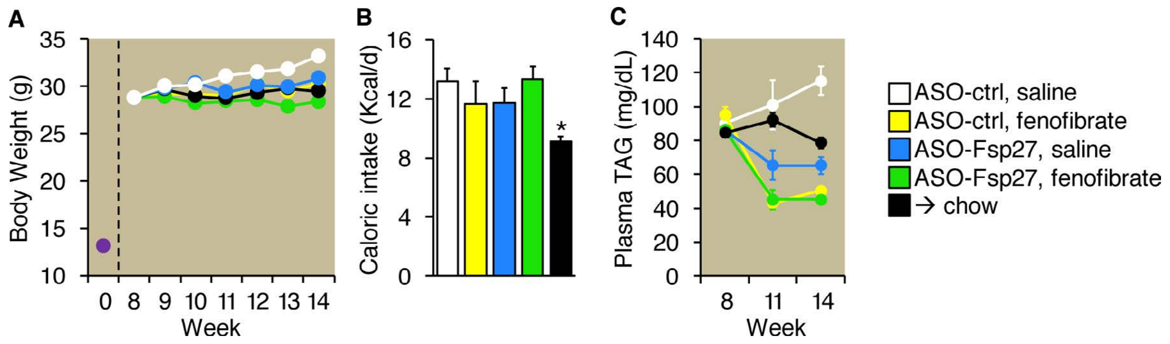

Supplemental Fig. S1. Fenofibrate- and ASO-Fsp27-dependent changes in body weight and plasma TAG are not the result of altered food intake. See Fig. 1A for details on the experimental timeline. (A) Body mass before the mice were fed NASH diet (purple dot), and during the last 6 weeks of the experiment, when they were treated with the drugs or switched to chow. (B) Caloric intake during the last week of the experiment. (C) Plasma TAG levels before mice were treated with drugs, and after 3 and 6 weeks of treatment.

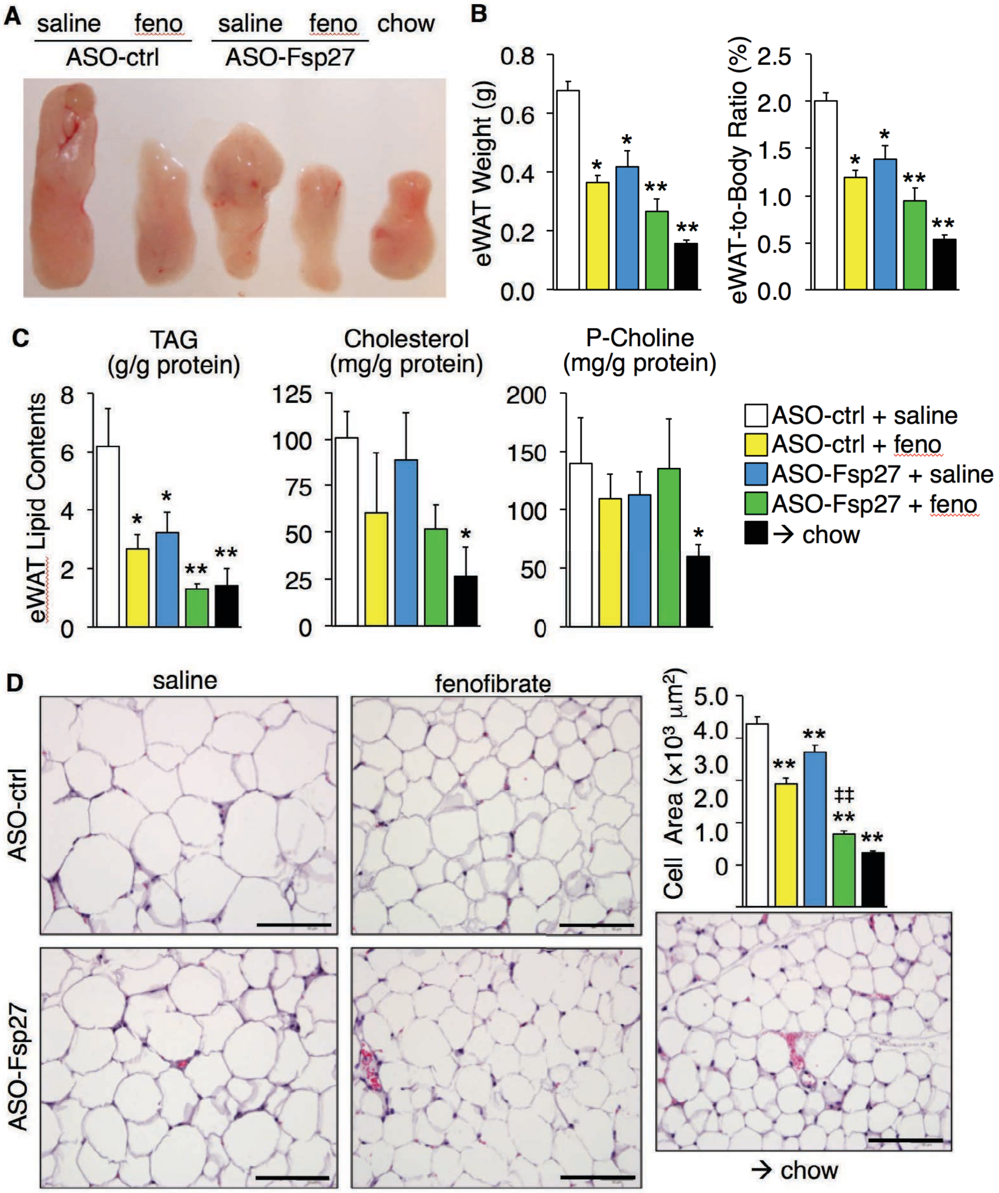

Supplemental Fig. S2. ASO-Fsp27 and fenofibrate synergistically reduce visceral adiposity in NASH diet-fed mice. (A) Representative macroscopic appearance of the left epididymal fat pad (eWAT) in each experimental group. (B) Absolute and relative weights of eWAT. (C) Lipid contents of eWAT. (D) Representative micrographs of hematoxylin and eosin-stained paraffin-embedded sections. Average cell areas were calculated from at least 500 cells in each experimental group using ImageJ. Data are shown as mean ± s.e.m. (*n*=7). **P*≤0.05, ***P*≤0.01, compared to [ASO-ctrl, saline]; ‡*P*≤0.05, significant interaction between ASO-Fsp27 and fenofibrate treatments.

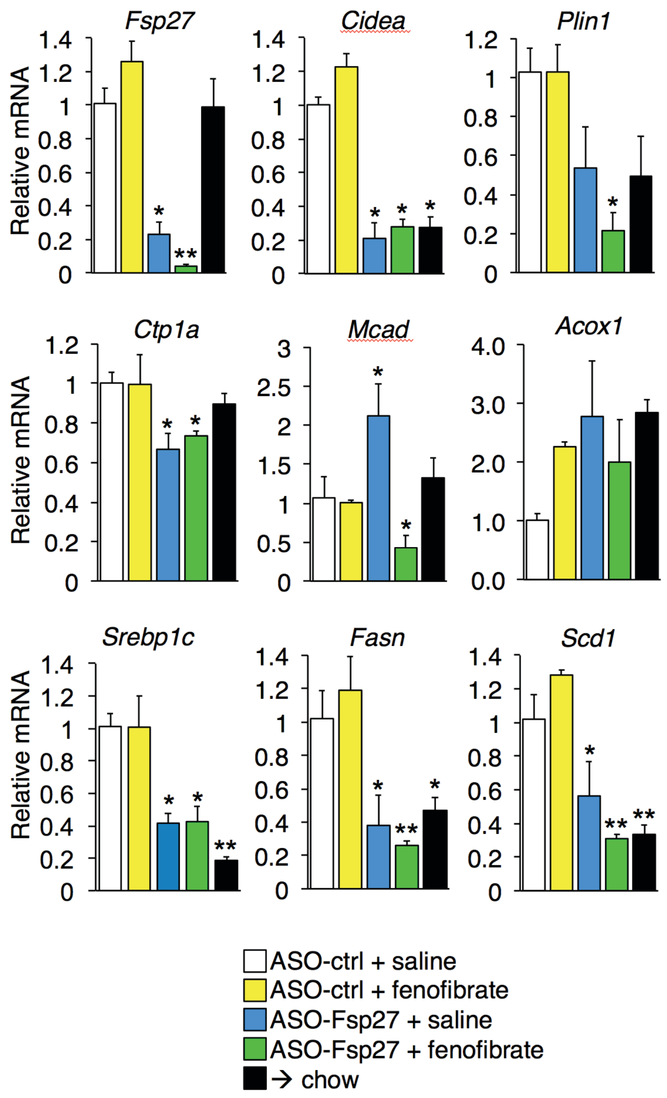

Supplemental Fig. S3. Relative expression of selected lipid-related transcripts in eWAT. Adipose tissue RNA contents were analyzed by qPCR. Data show the efficiency of *Fsp27* silencing, and the lack of induction of *bona fide* PPARα targets by fenofibrate. Data are shown as mean ± s.e.m. (n=5). **P*≤0.05, ***P*≤0.01, compared to [ASO-ctrl, saline]; ‡*P*≤0.05, significant interaction between ASO-Fsp27 and fenofibrate treatments.

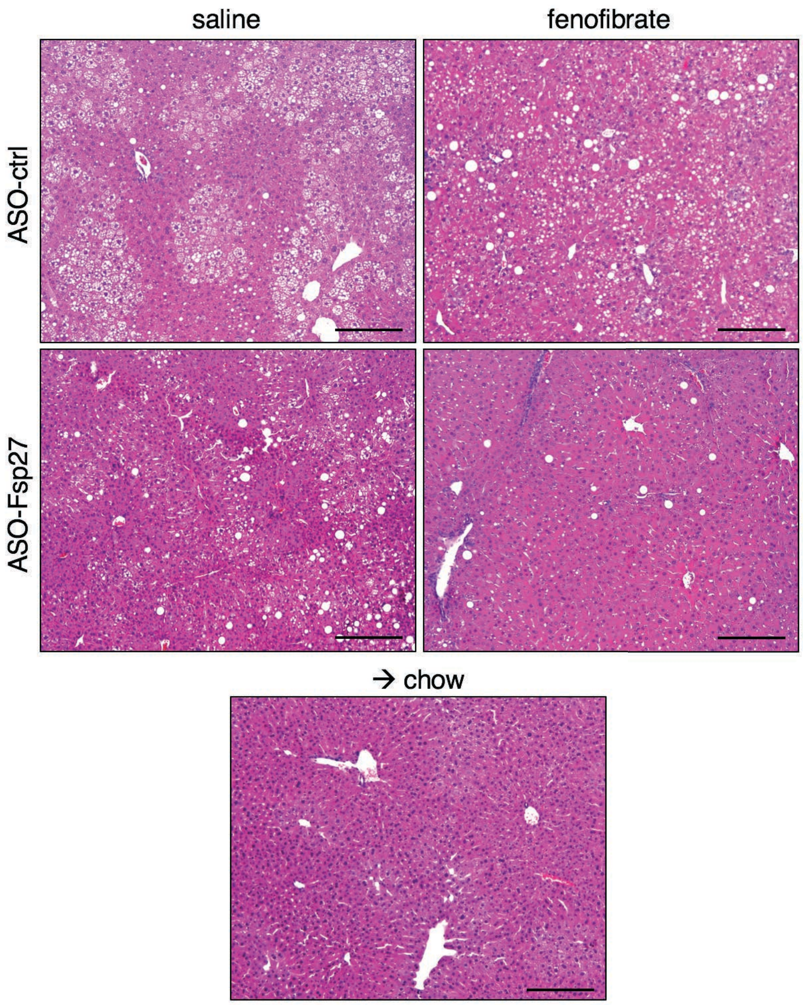

Supplemental Fig. S4. Improved hepatic histological appearance following combined ASO-Fsp27 and fenofibrate treatment. Representative hematoxylin and eosin staining of paraffin embedded sections. Scale bars represent 200 μm.

Antibody	Provider	Catalog #	Dilution
CIDEB	Abcam	ab58805	1:500
CPT1A	Proteintech	151841AP	1:1,000
MCAD	Abcam	ab110296	1:500
HMGCS2	Abcam	ab137043	1:2,000
FASN	Abcam	ab22759	1:1,000
SCD1	Abcam	ab19862	1:1,000
DGAT1	Novus	NB100-57086	1:2,000
CAT	Abcam	ab16731	1:2,000
SOD2	Abcam	ab13533	1:5,000
HMOX1	Abcam	ab13248	1:1,000
VCL	Santa Cruz	sc-7649	1:2,000
[rabbit IgG]-HRP	Santa Cruz	sc-2749	1:20,000
[mouse IgG]-HRP	Santa Cruz	sc-2318	1:10,000
[goat IgG]-HRP	Santa Cruz	sc-2352	1:5,000

Supplemental Table S1. *Antibodies used in this study.*
